# Supplementary material for: ACAD10 and ACAD11 enable mammalian 4-hydroxy acid lipid catabolism
Source: Nat Struct Mol Biol. 2025 Jun 19;32(9):1622–32. doi: 10.1038/s41594-025-01596-4 (PMC12440821; doi:10.1038/s41594-025-01596-4)
Supplement: Supplementary file 1 — Captions for Supplementary Tables 1–6 and Figs. 1 and 2. [file 41594_2025_1596_MOESM1_ESM.pdf]

nature structural & molecular biology

Supplementary information

https://doi.org/10.1038/s41594-025-01596-4

ACAD10 and ACAD11 enable mammalian 4-hydroxy acid lipid catabolism

In the format provided by the authors and unedited

Supplementary Information Table-of-Contents

Supplementary Tables Guide | List of supplementary tables for this manuscript

Supplementary Figure 1 | Differential scanning fluorimetry of purified recombinant ACAD11 constructs. Top left corner graph displays melt curve pattern for full-length wild-type ACAD11 (black line) relative to the melt curve patterns for kinase (blue line) or ACAD domain (red line) in isolation. ACAD11 mutant variant information is indicated above each graph. Interpretable melt curves for recombinant ACAD10 constructs could not be generated due to presence of co-purifying contaminants (Extended Data Fig. 1c).

Supplementary Figure 2 | Next-generation sequencing of deleterious indels at CRISPR/Cas9 target sites in Hepa1-6 KO cells

Supplementary Tables Guide

Table 1: Plasmids,

Table 2: gBLOCK sequences

Table 3: Primer sequences

Table 4: Lipidomic dMRM parameters

Table 5: Lipidomic standards

Table 6: 4-HA lactone precursors

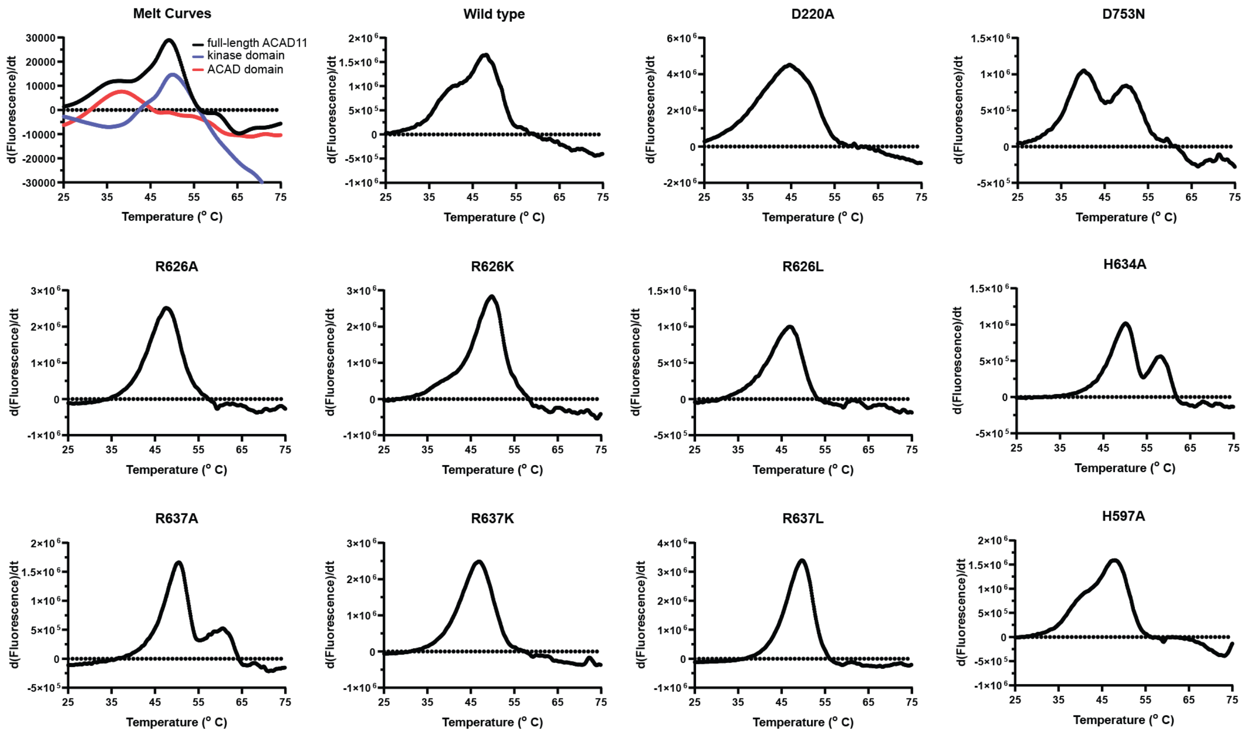

Supplementary Figure 1 | Differential scanning fluorimetry of purified recombinant ACAD11 constructs. Top left corner graph displays melt curve pattern for full-length wild-type ACAD11 (black line) relative to the melt curve patterns for kinase (blue line) or ACAD domain (red line) in isolation. ACAD11 mutant variant information is indicated above each graph. Interpretable melt curves for recombinant ACAD10 constructs could not be generated due to presence of co-purifying contaminants (Extended Data Fig. 1c).

<u>Hepa1-6 cell *Acad10* KO sequencing</u>

Clone	Sample	Total	sp1	#1-Indel	#1-Reads(%)	#2-Indel	#2-Reads(%)	#3-Indel	#3-Reads(%)
ACAD10 KO-a	Acad10 KO.2G2	4474	0 (0.0%)	1	2717 (60.7%)	-23	1285 (28.7%)	-20	456 (10.2%)
ACAD10 KO-b	Acad10 KO.3C6	1212	2 (0.2%)	-19	820 (67.7%)	1	388 (32.0%)	0	2 (0.2%)

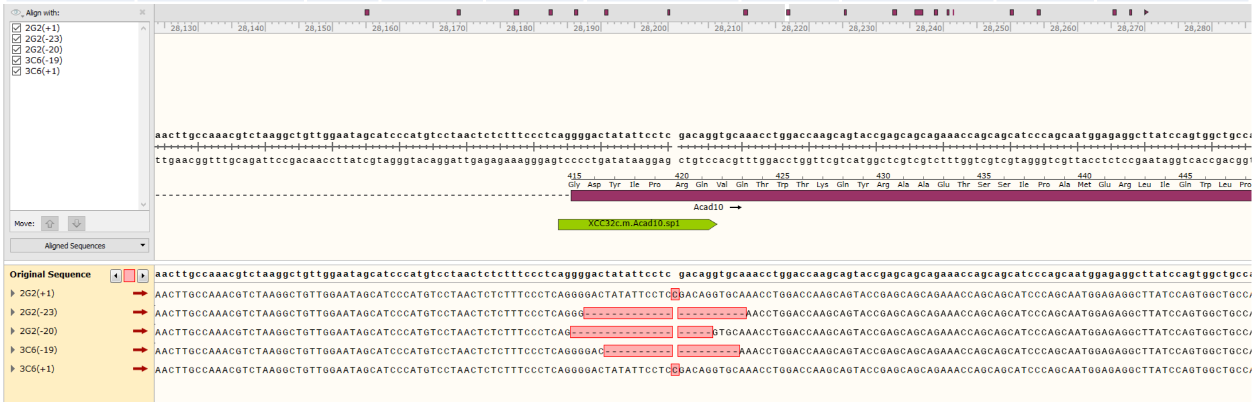

<u>Hepa1-6 cell *Acad11* KO sequencing</u>

Clone	Sample	Total	Sp2	#1-Indel	#1-Reads(%)	#2-Indel	#2-Reads(%)
ACAD11 KO-a	Acad11 KO.2G6	2476	9 (0.4%	-1	1210 (48.9%)	-14	1148 (46.4%)
ACAD11 KO-b	Acad11 KO.3D3	2125	2 (0.1%)	-5	1359 (64.0%)	-14	654 (30.8%)

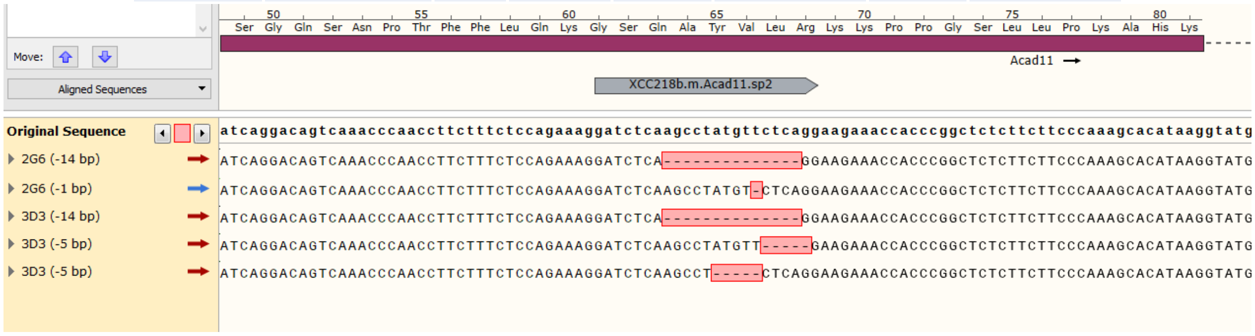

<u>Hepa1-6 cell *Acad10*/*Acad11* DKO sequencing</u>

Acad10 indel sequencing

Clone	Sample	Total	sp3	sp1	#1-Indel	#1-Reads(%)	#2-Indel	#2-Reads(%)	#3-Indel	#3-Reads(%)
DKO-a	DKO.2B4	1696	0 (0.0%)	0 (0.0%)	1	567 (33.4%)	-7	564 (33.3%)	-1	559 (33.0%)
DKO-b	DKO.3F8	3786	0 (0.0%)	0 (0.0%)	-2	1297 (34.3%)	-1	1281 (33.8%)	-10	1207 (31.9%)

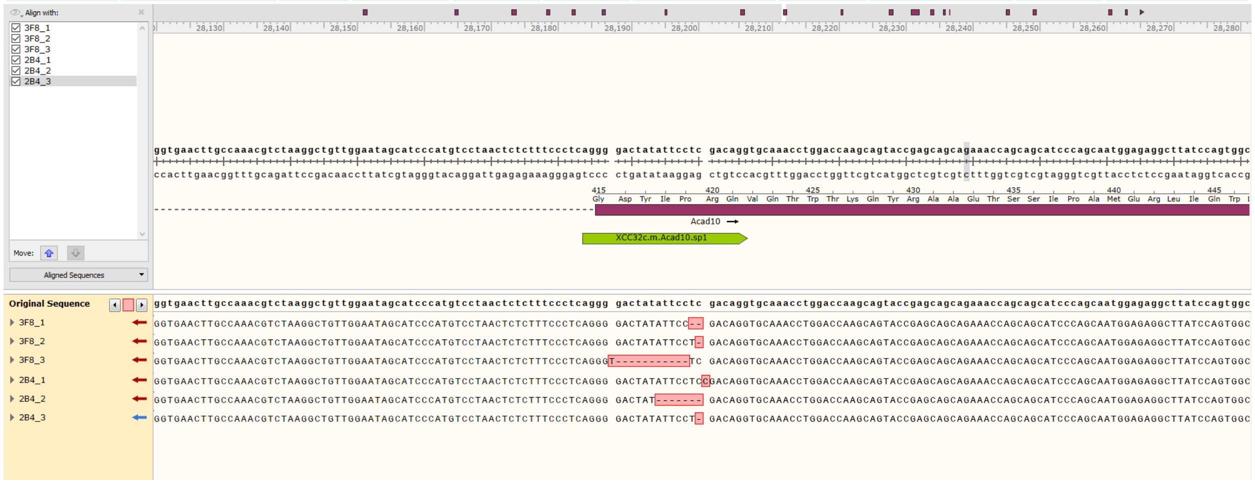

Acad11 indel sequencing

Clone	Sample	Total	sp2	sp5	#1-Indel	#1-Reads(%)	#2-Indel	#2-Reads(%)	#3-Indel	#3-Reads(%)
DKO-a	DKO.2B4	905	2 (0.2%)	883 (97.6%)	-5	579 (64.0%)	-14	271 (29.9%)	-6	23 (2.5%)
DKO-b	DKO.3F8	2423	4 (0.2%)	2327 (96.0%)	-5	1548 (63.9%)	-14	748 (30.9%)	-6	57 (2.4%)

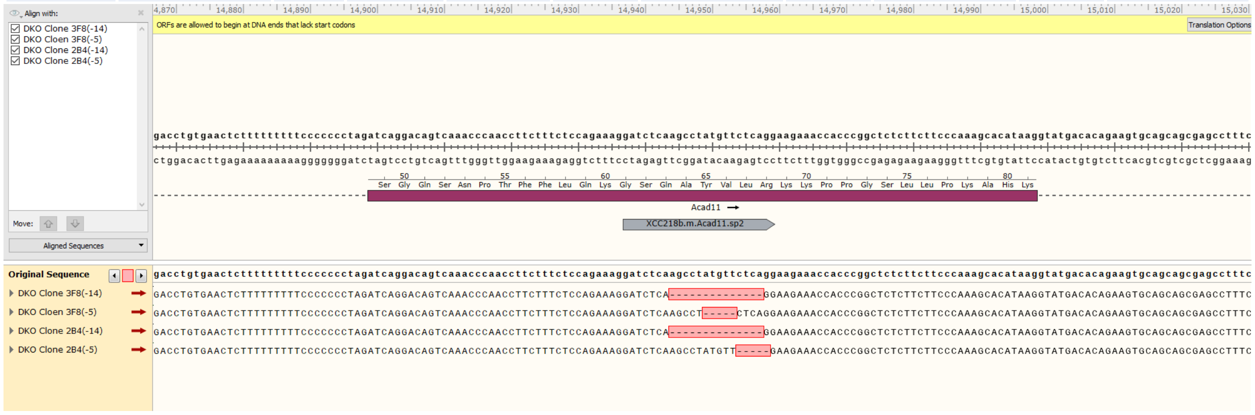

Supplementary Figure 2 | Next-generation sequencing of CRISPR/Cas9 target sites in Hepa1-6 KO cells. ACAD11 KO-b cell line was used to generate DKO-a and DKO-b cells.
