# Supplementary material for: ACAD10 and ACAD11 enable mammalian 4-hydroxy acid lipid catabolism
Source: Nat Struct Mol Biol. 2025 Jun 19;32(9):1622–32. doi: 10.1038/s41594-025-01596-4 (PMC12440821; doi:10.1038/s41594-025-01596-4)
Supplement: Supplementary file 2 — Reporting Summary [file 41594_2025_1596_MOESM2_ESM.pdf]

nature portfolio

Corresponding author(s): Pagliarini, David J. and Simcox, Judith A.

Last updated by author(s): 5/15/2025

Reporting Summary

Nature Portfolio wishes to improve the reproducibility of the work that we publish. This form provides structure for consistency and transparency in reporting. For further information on Nature Portfolio policies, see our Editorial Policies and the Editorial Policy Checklist.

Statistics

For all statistical analyses, confirm that the following items are present in the figure legend, table legend, main text, or Methods section.

n/a	Confirmed	
☐	☒	The exact sample size (*n*) for each experimental group/condition, given as a discrete number and unit of measurement
☐	☒	A statement on whether measurements were taken from distinct samples or whether the same sample was measured repeatedly
☐	☒	The statistical test(s) used AND whether they are one- or two-sided<br>*Only common tests should be described solely by name; describe more complex techniques in the Methods section.*
☐	☒	A description of all covariates tested
☒	☐	A description of any assumptions or corrections, such as tests of normality and adjustment for multiple comparisons
☐	☒	A full description of the statistical parameters including central tendency (e.g. means) or other basic estimates (e.g. regression coefficient) AND variation (e.g. standard deviation) or associated estimates of uncertainty (e.g. confidence intervals)
☐	☒	For null hypothesis testing, the test statistic (e.g. *F*, *t*, *r*) with confidence intervals, effect sizes, degrees of freedom and *P* value noted<br>*Give P values as exact values whenever suitable.*
☒	☐	For Bayesian analysis, information on the choice of priors and Markov chain Monte Carlo settings
☒	☐	For hierarchical and complex designs, identification of the appropriate level for tests and full reporting of outcomes
☐	☒	Estimates of effect sizes (e.g. Cohen's *d*, Pearson's *r*), indicating how they were calculated

Our web collection on statistics for biologists contains articles on many of the points above.

Software and code

Policy information about availability of computer code

Data collection	Electrophoresis gel images and Western blots were imaged using LI-COR Image Studio (v5.2.5). HPLC data was collected using Thermo Scientific Chromeleon console (v7.2.10). Plate reader data was collected using BioTek Gen5 software (v1.11.5). All LC-MS data was collected using the following commercial software: Thermo Scientific Xcalibur (v4.3 and v4.6), Agilent MassHunter Acquisition Method (v10.1)
Data analysis	Cryo-EM data was processed using cryoSPARC (v3.3.1), deepEMhancer (v0.14), COOT (v0.9.8), PHENIX (v1.20.1), and ChimeraX (v1.5). Molecular modeling simulations were performed using Gromacs (2022), VMD (1.9.4). Manual analysis of models was performed using PyMOL (v3.1.4) via the HandMOL VR interface. HPLC data was analyzed using Thermo Scientific Chromeleon console (v7.2.10) and Graphpad Prism (v10.0.3). Plate reader and other in vitro assay data was analyzed using Microsoft Excel or Graphpad Prism (v9.4.1 or v10.0.3), including all statistical analyses unless otherwise mentioned. Metabolomics and stable isotope tracing mass spectrometry data was processed in El-MAVEN (v0.12.0), Thermo Fisher Scientific Xcalibur (v4.3 and v4.6), and Thermo Scientific Tracefinder (v5.1) softwares. Mouse plasma mass spectrometry data was analyzed using the Agilent MassHunter Suite (v10.1). Fluorescence intensity profiles for microscopy were prepared using ImageJ software (v2.9.0/1.53t) and/or CellProfiler (v4.2.8). Quantitative PCR data was analyzed using Applied Biosystems QuantStudio Real-Time PCR software (v1.5.1). Organellar localization Pearson correlations were analyzed using R (v4.2.2).

For manuscripts utilizing custom algorithms or software that are central to the research but not yet described in published literature, software must be made available to editors and reviewers. We strongly encourage code deposition in a community repository (e.g. GitHub). See the Nature Portfolio guidelines for submitting code & software for further information.

Data

Policy information about availability of data

All manuscripts must include a data availability statement. This statement should provide the following information, where applicable:

- Accession codes, unique identifiers, or web links for publicly available datasets
- A description of any restrictions on data availability
- For clinical datasets or third party data, please ensure that the statement adheres to our policy

All enzyme assay, mouse physiology, microscopy, and mass spectrometry datasets used to generate figures are available in the Source Data files. Protein sequences used for mammalian homology analysis were obtained from the UniProt database (Taxon ID 40674). Raw mass spectrometry files were deposited to the MassIVE repository under the accession numbers MSV000097576 (in vitro enzyme assay and cell culture metabolomics) and MSV000097543 (mouse plasma lipidomics). The accession numbers for cryo-electron microscopy structures are PDB 8V3U and 8V3V. Raw microscopy and gel images are provided as Supplementary Information. Other relevant data reported in this work, including raw HPLC data files, are available from the corresponding author upon reasonable request.

Research involving human participants, their data, or biological material

Policy information about studies with human participants or human data. See also policy information about sex, gender (identity/presentation), and sexual orientation and race, ethnicity and racism.

Reporting on sex and gender	This study does not involve human participants or use human data.
Reporting on race, ethnicity, or other socially relevant groupings	This study does not involve human participants or use human data.
Population characteristics	This study does not involve human participants or use human data.
Recruitment	This study does not involve human participants or use human data.
Ethics oversight	This study does not involve human participants or use human data.

Note that full information on the approval of the study protocol must also be provided in the manuscript.

Field-specific reporting

Please select the one below that is the best fit for your research. If you are not sure, read the appropriate sections before making your selection.

☒ Life sciences ☐ Behavioural & social sciences ☐ Ecological, evolutionary & environmental sciences

For a reference copy of the document with all sections, see nature.com/documents/nr-reporting-summary-flat.pdf

Life sciences study design

All studies must disclose on these points even when the disclosure is negative.

Sample size	All experiments were performed in triplicate or more. No statistical approaches were used to determine sample size. Sample sizes chosen (3 or more samples) were rational as they allowed for appropriate statistical testing.
Data exclusions	Lipid measurements that were poorly detected (not detected in >40% samples or signal below background levels) and outlier lipid measurements (two standard deviations outside of the group mean) were excluded from final lipidomics analysis.
Replication	Three or more technical or biological replicates were used in each experiment when applicable. All attempts at replicating experimental results were successful.
Randomization	Measurements were made quantitatively and automated when possible to mitigate Investigator bias. When necessary, injection order of HPLC and MS samples was randomized to limit bias or noise introduced by technical factors (i.e., running all technical or biological replicates of a given condition consecutively).
Blinding	For animal work, Investigators were only blinded to genotype of mice during mouse euthanization, sample collection, and subsequent sample analysis. For in vitro enzymology and cell culture work, blinding was not possible since the experimental setup requires knowledge of the sample identities.

Reporting for specific materials, systems and methods

We require information from authors about some types of materials, experimental systems and methods used in many studies. Here, indicate whether each material, system or method listed is relevant to your study. If you are not sure if a list item applies to your research, read the appropriate section before selecting a response.

Materials & experimental systems

n/a	Involved in the study
☐	☒ Antibodies
☐	☒ Eukaryotic cell lines
☒	☐ Palaeontology and archaeology
☐	☒ Animals and other organisms
☒	☐ Clinical data
☒	☐ Dual use research of concern
☒	☐ Plants

Methods

n/a	Involved in the study
☒	☐ ChIP-seq
☒	☐ Flow cytometry
☒	☐ MRI-based neuroimaging

Antibodies

Antibodies used

Western blot Primary antibody: Rabbit anti-GFP (1:1000; Abcam; ab6556)

Western blot Secondary antibody: IRDye 680RD Goat anti-Rabbit IgG SeconWdary Antibody (1:5000; Licor Bio; 926-68071)

Imaging Primary antibodies: Mouse anti-FLAG M2 (Sigma; F1804); rabbit anti-PEX14 (1:500; EMD Millipore; ABC142); chicken anti-HSP60 (1:500; EnCor Biotechnology; CPCA-HSP60).

Imaging Secondary antibodies: Goat anti-Mouse IgG (H+L) Cross-Adsorbed Secondary Antibody, Alexa Fluor 488 (1:500; Thermo Scientific; A-11001); Goat anti-Rabbit IgG (H+L) Cross-Adsorbed Secondary Antibody, Alexa Fluor 568 (1:500; Thermo Scientific; A-11011); Goat anti-Chicken IgY (H+L) Cross-Adsorbed Secondary Antibody, Alexa Fluor Plus 647 (1:500; Thermo Scientific; A32933)

Validation

The rabbit anti-GFP (Abcam; ab6556) was validated by the manufacturer according to their website. Antibody was determined to be specific to all variants of Aequorea victoria GFP (UniProt ID: P42212; Molecular weight: 27kDa).

The mouse anti-FLAG M2 (Sigma; F1804) was validated by the manufacturer according to their website. Specificity was determined by detection of target protein on a Western blot from an E. coli, plant, or mammalian crude cell lysate. Sensitivity was determined by dot blot, detecting as little as 2 ng of target protein.

The rabbit anti-PEX14 (EMD Millipore; ABC142) was validated by the manufacturer according to their website. Specificity was determined by detection of target protein on a Western blot from NIH/3T3 cell lysate and human liver lysate.

The chicken anti-HSP60 (EnCor Biotechnology; CPCA-HSP60) was validated by the manufacturer according to their website. Specificity was determined by detection of target protein on a Western blot from SH-SY5Y and HeLa cells.

Eukaryotic cell lines

Policy information about cell lines and Sex and Gender in Research

Cell line source(s)

Hepa1-6 cells (CRL-1830), U-2 OS cells (HTB-96), and COS7 cells (CRL-1651) were purchased from the American Type Culture Collection (ATCC). Hepa1-6 parental cells were used to generate single and double knockouts of ACAD10 and/or ACAD11 via CRISPR/Cas9 technology (performed by the Genome Engineering and Stem Cell Center (GESC) at Washington University in St. Louis).

Authentication

Hepa1-6 KO cell lines were authenticated by GESC by STR analysis and next-generation sequencing of amplicons of the targeted regions. After transfection of sgRNAs and clonal selection, KO clones were chosen for this study if all indels at the select target sites were predicted to introduce premature stop codons and no wild-type alleles were detected. Next-generation sequencing results for all KO lines are included in the Supplementary Information file.

Mycoplasma contamination

All cell lines used in this study tested negative for mycoplasma contamination.

Commonly misidentified lines
(See ICLAC register)

No commonly misidentified lines were utilized in this study.

Animals and other research organisms

Policy information about studies involving animals; ARRIVE guidelines recommended for reporting animal research, and Sex and Gender in Research

Laboratory animals

Animal work was conducted with cohorts of mice in the C57BL/6NJ mixed background. In vivo experimental groups were 3 months old, 4-5 months old, or 8 months old and included n = 3-10 mice. All experimental mice were evaluated with litter-mate controls.

Wild animals

This study did not utilize wild animals.

Reporting on sex	Both male and female mice were evaluated in all in vivo experiments.
Field-collected samples	This study did not utilize field-collected samples.
Ethics oversight	All animal experiments were approved by the Institutional Animal Care and Use Committee of the College of Agricultural and Life Sciences at the University of Wisconsin-Madison.

Note that full information on the approval of the study protocol must also be provided in the manuscript.

Plants

Seed stocks	Plant models were not used in this study
Novel plant genotypes	n/a
Authentication	n/a
