# Supplementary material for: ACAD10 and ACAD11 enable mammalian 4-hydroxy acid lipid catabolism
Source: Nat Struct Mol Biol. 2025 Jun 19;32(9):1622–32. doi: 10.1038/s41594-025-01596-4 (PMC12440821; doi:10.1038/s41594-025-01596-4)
Supplement: Supplementary file 10 — Image source data. [file 41594_2025_1596_MOESM10_ESM.pdf]

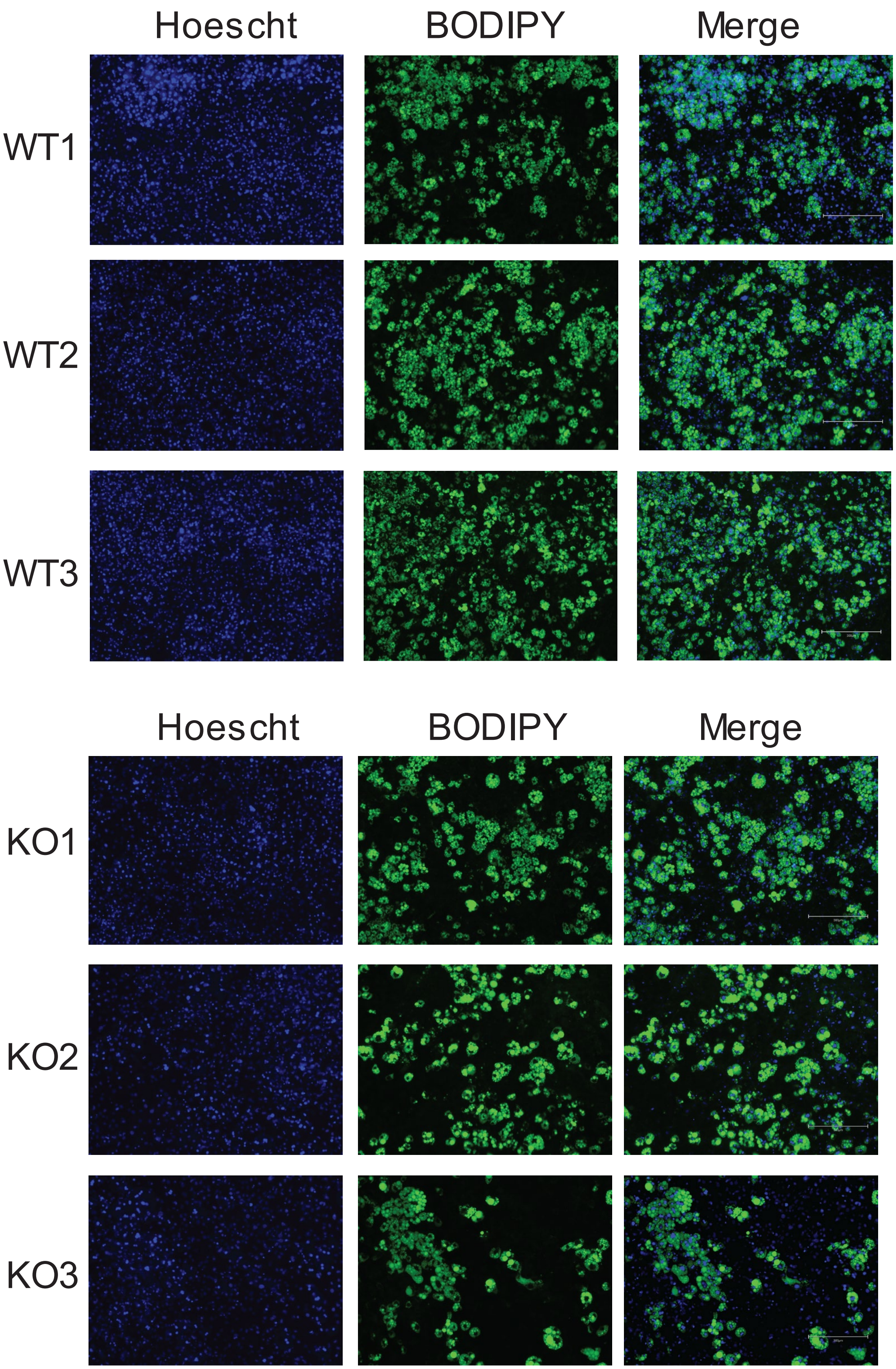

Representative raw fluorescence microscopy images of female WT and *Acad11* pre-adipocytes after eight days of differentiation *ex vivo*. "WT2" and "KO2" images are representative images shown in Fig. 5k.

ACAD11 WT1 – Tech Rep 1

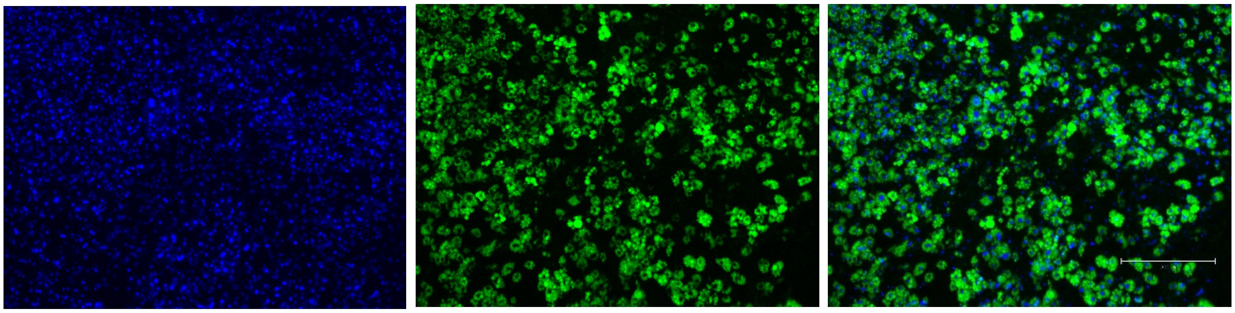

ACAD11 WT1 – Tech Rep 2

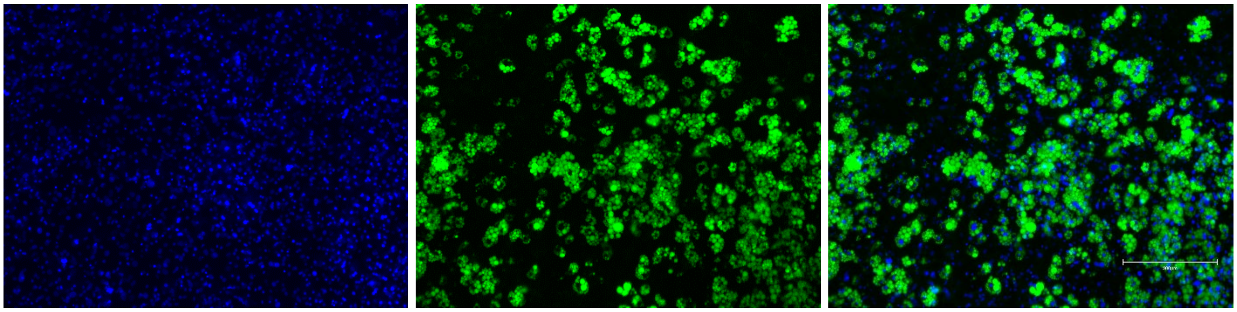

ACAD11 WT2 – Tech Rep 1

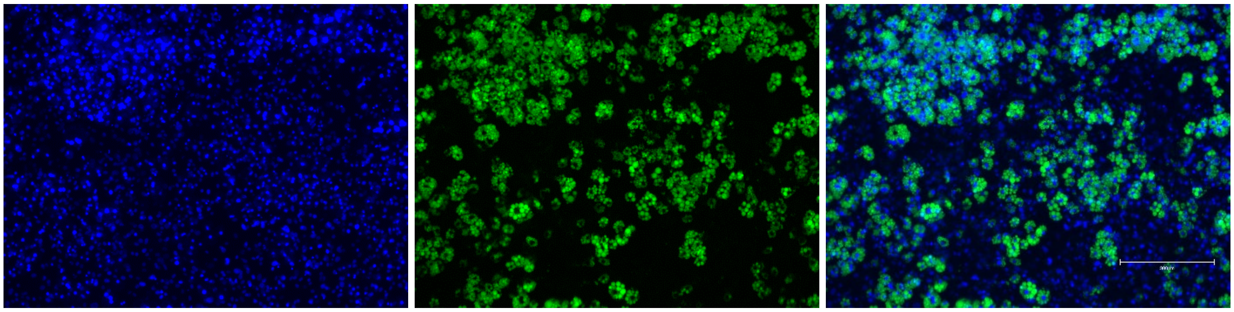

ACAD11 WT2 – Tech Rep 2

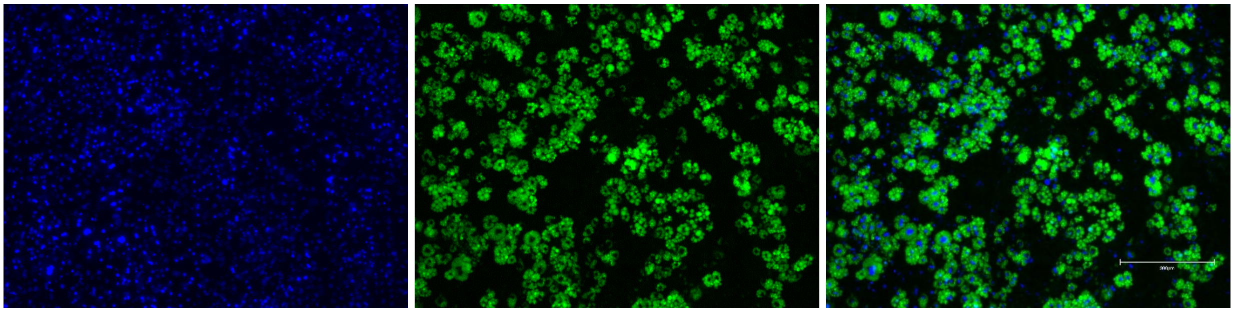

ACAD11 WT3 – Tech Rep 1

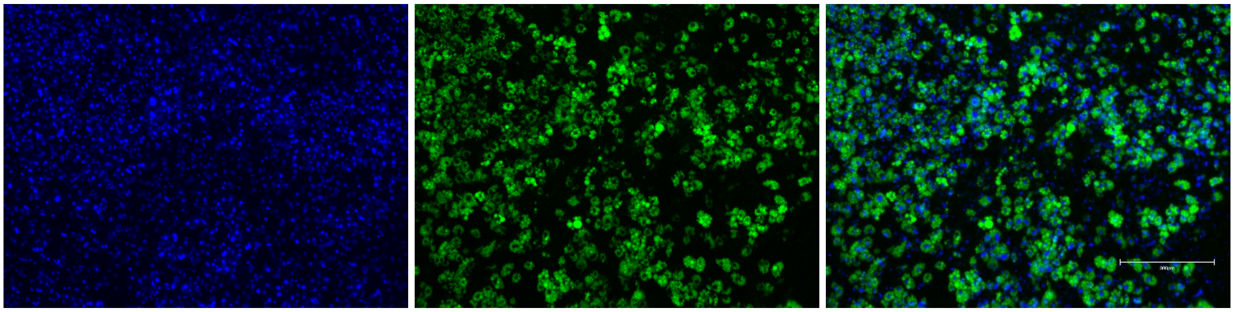

ACAD11 WT3 – Tech Rep 2

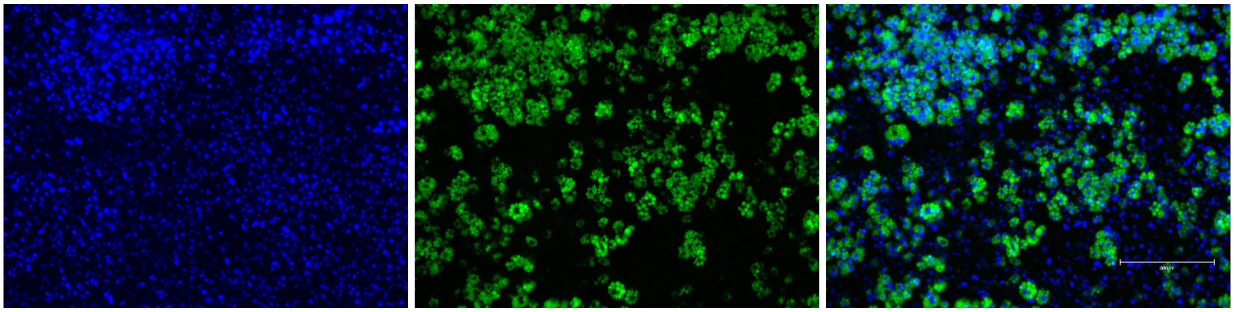

ACAD11 WT3 – Tech Rep 3

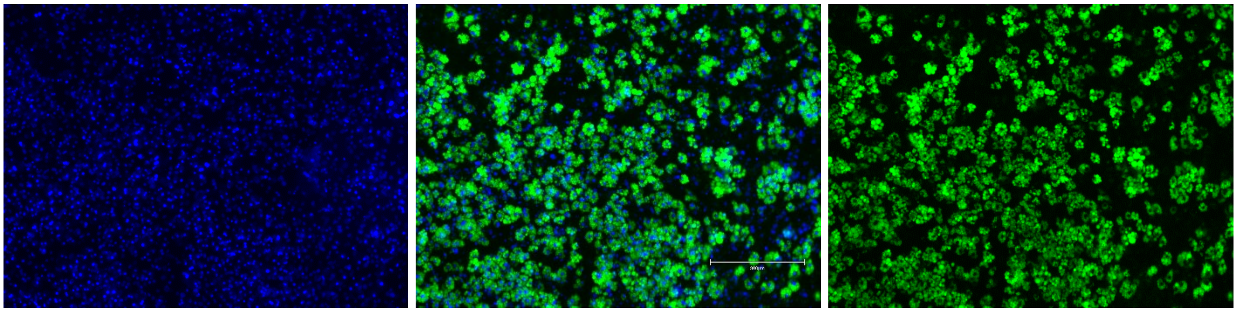

ACAD11 KO1 – Tech Rep 1

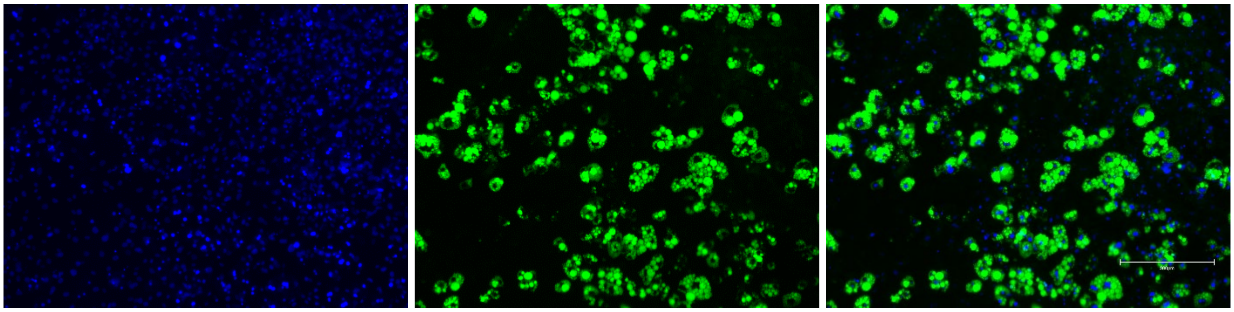

ACAD11 KO1 – Tech Rep 2

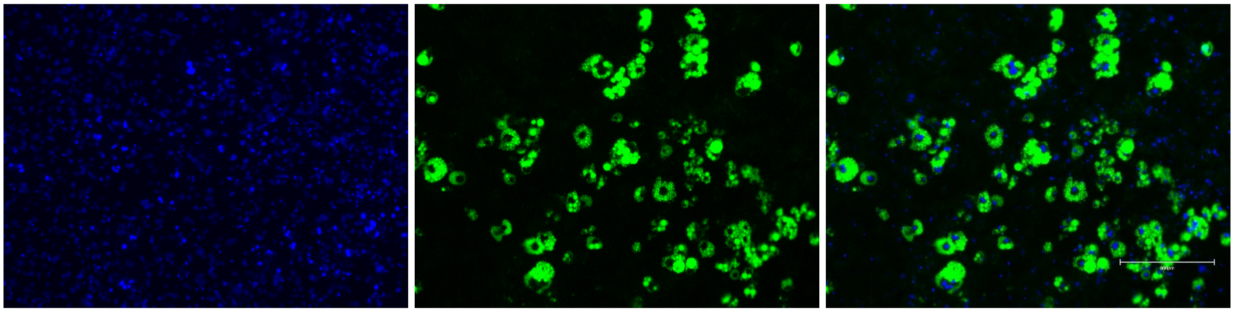

ACAD11 KO1 – Tech Rep 3

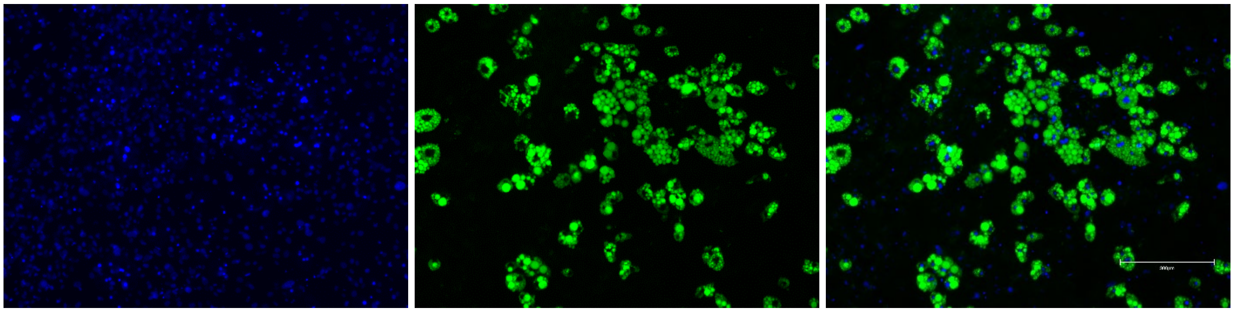

ACAD11 KO2 – Tech Rep 1

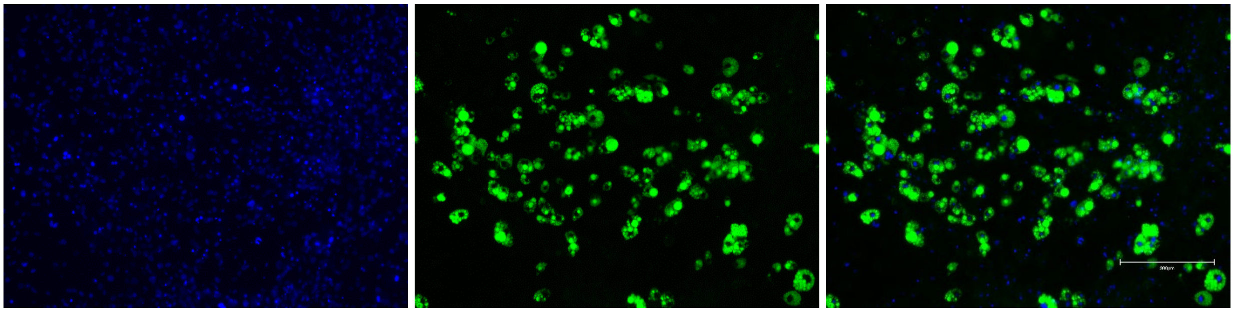

ACAD11 KO2 – Tech Rep 2

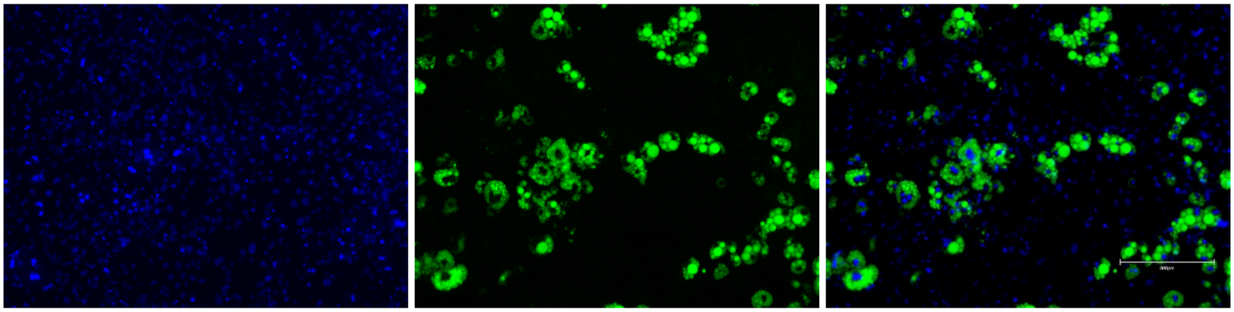

ACAD11 KO2 – Tech Rep 3

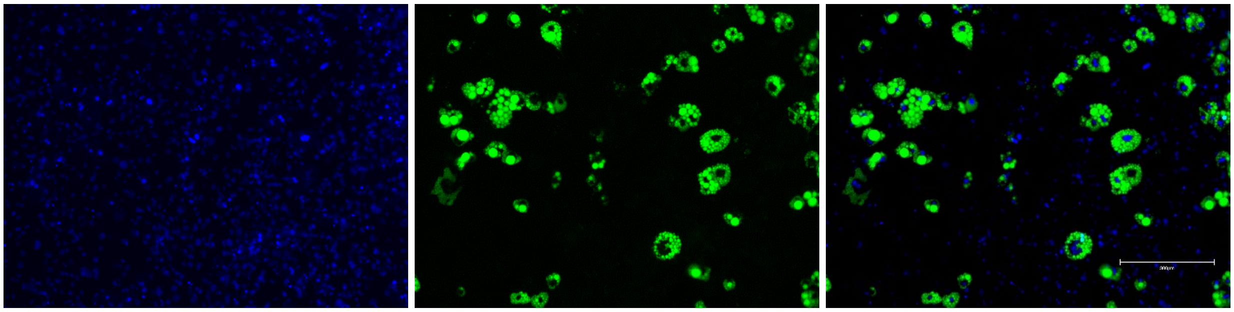

ACAD11 KO3 – Tech Rep 1

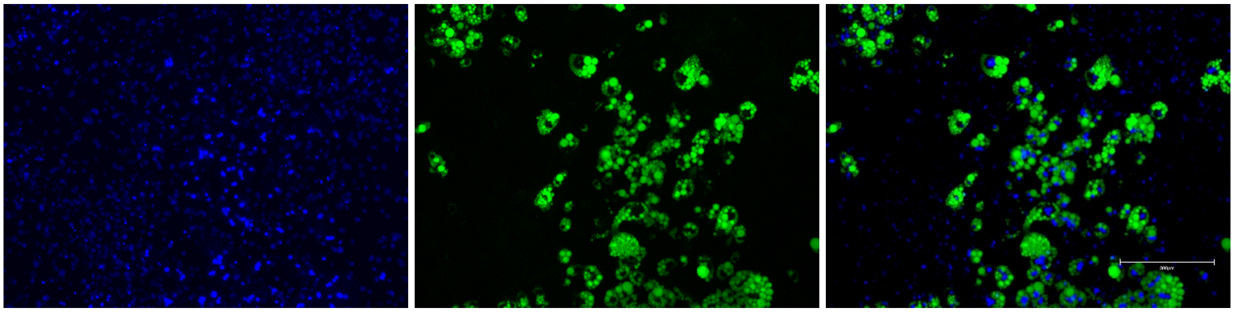

ACAD11 KO3 – Tech Rep 2

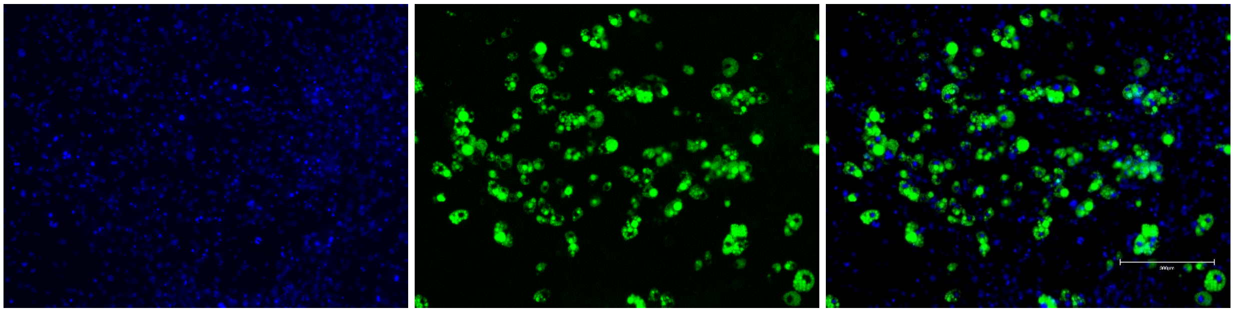

ACAD11 KO3 – Tech Rep 3

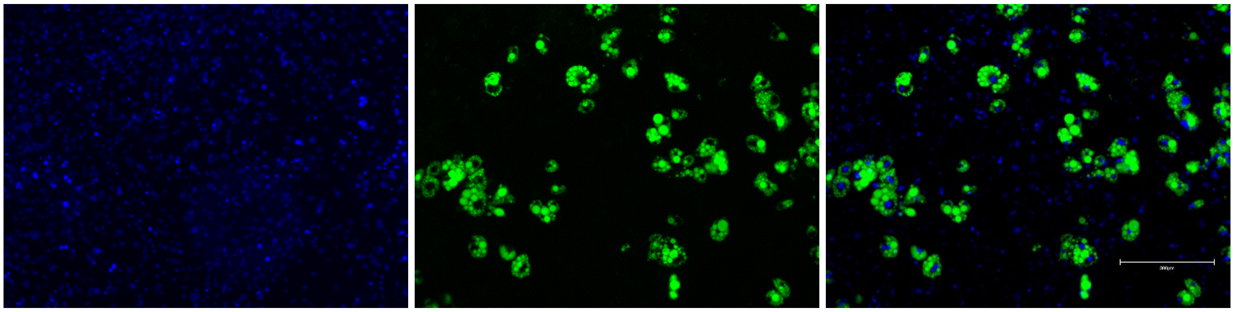

Raw fluorescence microscopy images of female WT and *Acad11* pre-adipocytes after eight days of differentiation *ex vivo* that were used for lipid droplet quantity, lipid droplet size, and nuclei size quantification in Fig. 5l-n.
