# Supplementary figures and images for: ACAD10 and ACAD11 enable mammalian 4-hydroxy acid lipid catabolism
Source: Nat Struct Mol Biol. 2025 Jun 19;32(9):1622–32. doi: 10.1038/s41594-025-01596-4 (PMC12440821; doi:10.1038/s41594-025-01596-4)

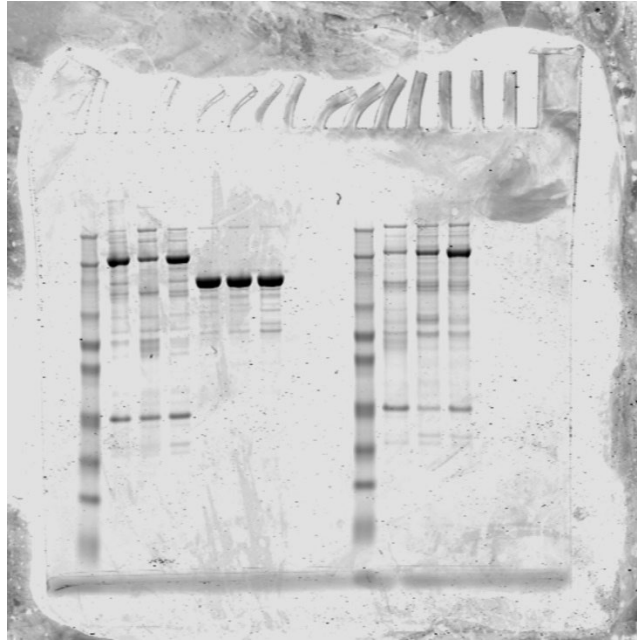

Raw SDS-PAGE gel image of purified recombinant mouse ACAD10 and ACAD11 (**Extended Data Fig. 1c**)

Supplement: Supplementary file 17 — Uncropped gel source data. [file 41594_2025_1596_MOESM17_ESM.pdf]
