# Supplementary material for: ACAD10 and ACAD11 enable mammalian 4-hydroxy acid lipid catabolism
Source: Nat Struct Mol Biol. 2025 Jun 19;32(9):1622–32. doi: 10.1038/s41594-025-01596-4 (PMC12440821; doi:10.1038/s41594-025-01596-4)
Supplement: Supplementary file 18 — Uncropped gel source data. [file 41594_2025_1596_MOESM18_ESM.pdf]

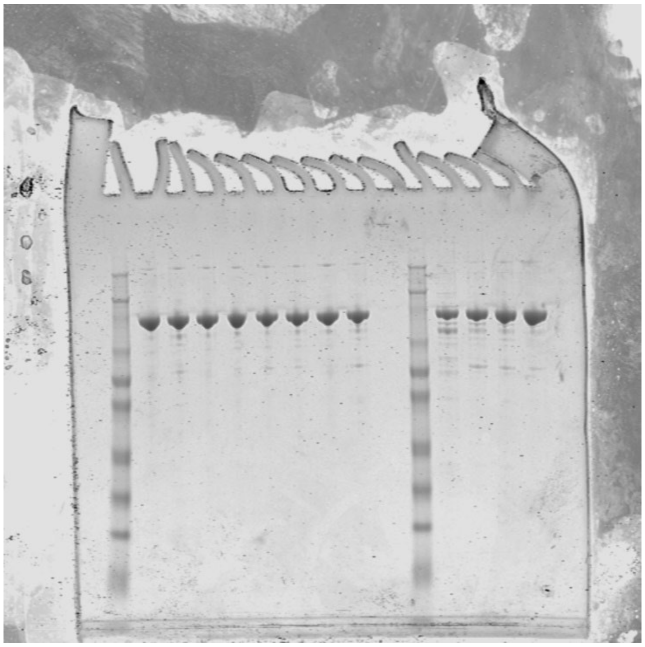

Raw SDS-PAGE gel image of purified recombinant mouse ACAD11 ACAD domain mutants
(**Extended Data Fig. 5d**)
